# P02.109. Stress management counseling in primary care: results of a national study

**DOI:** 10.1186/1472-6882-12-S1-P165

**Published:** 2012-06-12

**Authors:** A Nerurkar, A Bitton, R Davis, R Phillips, G Yeh

**Affiliations:** 1 Beth Israel Deaconess Medical Center; Harvard Medical School, Boston, USA; 2 Brigham & Women's Hospital, Boston, USA

## Purpose

Evidence suggests an association between stress management and improvements in conditions commonly seen in primary care. Few data, however, are available on the characteristics of primary care visits that include stress management counseling.

## Methods

Using cross-sectional data from the 2006-2009 National Ambulatory Medical Care Surveys (n=123,192), we examined the prevalence of physician counseling about stress in the primary care setting. We used logistic regression analyses to identify characteristics (patient sociodemographics, health status, diagnoses, and health utilization) associated with visits offering stress management counseling (n=1,020) and compared these to visits without stress management counseling (n=33,045).

## Results

Physicians offered stress management counseling in 39.3 million visits, representing 3.0% of all primary care visits. Of visits with some type of counseling provided (n=8,527), counseling about stress management was the least common (12.2%) followed by physical activity (30.3%), nutrition (22.2%), weight reduction (21.5%) and tobacco cessation (13.7%). Patients counseled about stress were younger compared to those who were not counseled (mean age 51.4 vs. 54.5). After adjusting for patient age, sex and race, visits addressing a flare of a chronic problem were more likely to be associated with counseling (aOR 1.45 [1.06, 1.98] compared to a stable chronic problem). Longer visits and those for more chronic conditions had a greater likelihood for stress management counseling (aOR2.07 [1.60, 2.66] for 21-40 minute visits and aOR1.76 [1.17, 2.63] for visits >=40 minutes, compared to visits ≤10 minutes; aOR1.75 [1.34, 2.29] for 1-2 conditions, aOR2.01 [1.35, 2.98] for 3-4 conditions, and aOR3.45 [2.30, 5.18] for ≥5 conditions, compared to no conditions). Visits for depression were associated with physician counseling about stress (aOR 3.87 [2.87, 5.20]).

## Conclusion

Stress management counseling is uncommon in the primary care setting. When offered, it is associated with longer visits for complex patients who have numerous, chronic medical conditions. This may reflect potential missed opportunities.

